# Determining the Prevalence and Incidence of SARS-CoV-2 Infection in Prisons in England: Protocol for a Repeated Panel Survey and Enhanced Outbreak Study

**DOI:** 10.2196/30749

**Published:** 2022-01-12

**Authors:** Emma Plugge, Danielle Burke, Maciej Czachorowski, Kerry Gutridge, Fiona Maxwell, Nuala McGrath, Oscar O'Mara, Eamonn O'Moore, Julie Parkes

**Affiliations:** 1 University of Southampton Southampton United Kingdom; 2 Public Health England London United Kingdom; 3 Office for National Statistics London United Kingdom; 4 University of Manchester Manchester United Kingdom; 5 Ministry of Justice London United Kingdom

**Keywords:** COVID-19, epidemiology, prison, outbreak, testing, health inequalities, SARS-CoV-2

## Abstract

**Background:**

There are over 80,000 people imprisoned in England and Wales in 117 prisons. The management of the COVID-19 pandemic presents particular challenges in this setting where confined, crowded, and poorly ventilated conditions facilitate the rapid spread of infectious diseases.

**Objective:**

The COVID-19 in Prison Study aims to examine the epidemiology of SARS-CoV-2 in prisons in England in order to inform public health policy and practice during the pandemic and recovery. The primary objective is to estimate the proportion of positive tests of SARS-CoV-2 infection among residents and staff within selected prisons. The secondary objectives include estimating the incidence rate of SARS-CoV-2 infection and examining how the proportion of positive tests and the incidence rate vary among individual, institutional, and system level factors.

**Methods:**

Phase 1 comprises a repeated panel survey of prison residents and staff in a representative sample of 28 prisons across England. All residents and staff in the study prisons are eligible for inclusion. Participants will be tested for SARS-CoV-2 using a nasopharyngeal swab twice (6 weeks apart). Staff will also be tested for antibodies to SARS-CoV-2. Phase 2 focuses on SARS-CoV-2 infection in prisons with recognized COVID-19 outbreaks. Any prison in England will be eligible to participate if an outbreak is declared. In 3 outbreak prisons, all participating staff and residents will be tested for SARS-CoV-2 antigens at the following 3 timepoints: as soon as possible after the outbreak is declared (day 0), 7 days later (day 7), and at day 28. They will be swabbed twice (a nasal swab for lateral flow device testing and a nasopharyngeal swab for polymerase chain reaction testing). Testing will be done by external contractors. Data will also be collected on individual, prison level, and community factors. Data will be stored and handled at the University of Southampton and Public Health England. Summary statistics will summarize the prison and participant characteristics. For the primary objective, simple proportions of individuals testing positive for SARS-CoV-2 and incidence rates will be calculated. Linear regression will examine the individual, institutional, system, and community factors associated with SARS-CoV-2 infection within prisons.

**Results:**

The UK Government’s Department for Health and Social Care funds the study. Data collection started on July 20, 2020, and will end on May 31, 2021. As of May 2021, we had enrolled 4192 staff members and 6496 imprisoned people in the study. Data analysis has started, and we expect to publish the initial findings in summer/autumn 2021. The main ethical consideration is the inclusion of prisoners, who are vulnerable participants.

**Conclusions:**

This study will provide unique data to inform the public health management of SARS-CoV-2 in prisons. Its findings will be of relevance to health policy makers and practitioners working in prisons.

**International Registered Report Identifier (IRRID):**

DERR1-10.2196/30749

## Introduction

On March 11, 2020, the World Health Organization (WHO) declared a global pandemic of COVID-19, an illness caused by infection with SARS-CoV-2. By May 26, 2021, there were over 167 million confirmed infections and over 3.4 million deaths globally [1]. The pandemic has affected the United Kingdom, and over 1 million people have now tested positive for SARS-CoV-2 [2]. The pandemic has resulted in considerable adverse health, economic, and social consequences, with disproportionate impacts on Black, Asian, and minority ethnic groups and poorer communities [3].

The management of the COVID-19 pandemic presents particular challenges in the prison setting, and currently, over 80,000 people are imprisoned in England and Wales in 117 prisons [4]. Outbreaks of infectious diseases are not uncommon in prison settings where the confined, crowded, and poorly ventilated conditions facilitate the rapid spread of infectious diseases [5]. Given emerging evidence that SARS-CoV-2 is transmitted easily and is much more likely to spread in closed settings [6], the potential for rapid “explosive” COVID-19 outbreaks within prisons is considerable. The consequences for the imprisoned people (residents) are likely to be serious given the high proportion of individuals with risk factors for serious disease and death. The prevalence rates of chronic liver disease, lung disease, obesity, and other noncommunicable diseases are likely to be higher in prison populations [7,8]. Furthermore, the proportion of “older” people in prisons is rising rapidly, and imprisoned people are more likely to come from an ethnic minority [9]. Both these are important emerging risk factors for severe disease.

COVID-19 outbreaks are of importance to not only prison residents and staff, but also the wider community, as prisons are not sealed off from the rest of society. Staff and professional and social visitors (such as lawyers and families of residents) enter and leave daily. Residents leave to attend court hearings or hospital appointments, and they might return to a different prison. Moreover, newly imprisoned people and those transferred from other prisons enter many prisons daily. Thus, there is a high risk of the introduction of SARS-CoV-2 into prisons and its transmission among residents, staff, and visitors. There is potential for onward transmission to the community by staff and visitors, and by people who are released. The risks of “feeding and seeding” outbreaks in prisons through the in and out movements of staff, residents, and others are considerable [10], and the WHO has asserted that prisons should be an integral part of the public health response to COVID-19 [11].

In England, 3 key organizations (Her Majesty’s Prison and Probation Service [HMPPS], National Health Service [NHS], and Public Health England [PHE]) work together to tackle COVID-19 in prisons, implementing measures across all prisons to save lives, protecting the NHS by reducing the number of people requiring specialist care in community-based hospitals, and enabling the continued operation of the prison estate. The implementation of a restricted prison regime and “compartmentalization” of residents have been key elements in preventing the spread of SARS-CoV-2 [10]. The restricted prison regime, instituted by HMPPS on March 24, 2020, resulted in the cessation of social visits, face-to-face education provision, training and employment activities (except for essential workers), access to gyms, and religious association. Time out of prison cells for residents was also limited to as little as the statutory minimum of an hour a day. There were restrictions on the number of people unlocked and number of people in exercise yards at any one time, thus enabling the enforcement of social distancing of 2 m for staff and residents. “Compartmentalization” has been implemented both between and within prisons. Between prison transfers have been minimized to reduce the risk of “seeding” infections into prisons. Within each prison, “cohorting” strategies have been developed to prevent the spread of infection among residents and to protect the most vulnerable. Shielding units have been established to isolate those who meet the criteria for extremely vulnerable or vulnerable [9]. These units have enhanced the levels of biosecurity, including dedicated staff. Protective isolation units accommodate known or probable COVID-19 cases in, primarily, single-cell accommodation. Reverse cohorting units enable the quarantining of newly imprisoned people for a period of up to 14 days (upper limit of the known incubation period for SARS-CoV-2) before they enter the general population.

Emerging data suggest that these strategies have been effective in the first wave of the pandemic [10], but outbreaks involving prisons occurred when community prevalence was low and prisons moved into the “recovery” period. In this period, the restricted regime was eased, with population flows into and out of prisons increasing as courts opened up [12]. Social and legal visits were reintroduced, and activities in the prison, such as exercise and education provision, increased. While the risk of incursion of infection into prisons (via infected staff in particular) is likely to be related to the community incidence, the risk of outbreaks will continue as long as the virus circulates. Closed settings, such as prisons, experience outbreaks even when the wider community prevalence is low. The opening up of the prison regime led to more interactions and therefore a greater transmission risk, creating a period of risk for the prison system, where the potential for new and large COVID-19 outbreaks might increase considerably.

There is evidence of an increase in mental distress in the community attributable to COVID-19 [13]. Concerns have grown about the impact of prison regime restrictions, which were introduced to prevent the spread of COVID-19, on the mental well-being of imprisoned people. Staff have also faced considerable work pressures. Some staff have been ill with COVID-19, vulnerable staff have been shielding, and most staff have faced additional care responsibilities for imprisoned people, peers, families, and friends. As a result of high staff absence, the whole workforce is impacted, with the remaining staff struggling to ensure a decent and safe regime. This has consequences for the health of all those who live and work in prisons. There have been no studies examining the effects of COVID-19 on the mental health of these populations, although anecdotal reports include both positive impacts, such as an improved sense of safety, and many negative impacts, such as increased isolation, self-harm, and fear of the disease. Health care data from 31 prisons suggested an early reduction in self-harm [14], while a report on 3 women’s prisons suggested an increase in self-harm [15]. Further information on resident and staff mental well-being will complement the data on the epidemiology of SARS-CoV-2 infection in prisons, providing an additional dimension to inform policy and practice in managing COVID in prisons during the pandemic.

The COVID-19 in Prison Study aims to examine the epidemiology of SARS-CoV-2 in prisons in England in order to inform public health policy and practice during the pandemic and the recovery period.

The primary objective is to estimate the proportion of positive tests of SARS-CoV-2 infection among residents and staff within selected prisons. The secondary objectives are to (1) estimate the incidence rate of SARS-CoV-2 infection separately in residents and staff within selected prisons; (2) examine how the proportion of positive tests and the incidence rate vary among individual, institutional, and system level factors; (3) estimate the proportion of positive tests of antibodies (immunoglobulin G) to SARS-CoV-2 in prison staff; (4) characterize viral strains by genomic analysis; (5) examine the mental well-being of residents and staff in prisons; and (6) examine the sensitivity and specificity of the Innova lateral flow test in prison outbreaks.

This new knowledge will be used to inform decisions regarding the effectiveness of existing strategies to mitigate the spread of SARS-CoV-2 between and within prisons and to help develop effective recovery strategies within the prison estate in England.

## Methods

### Study Design

Phase 1 is a repeated panel survey of prison residents and staff in a sample of 28 prisons across England. The survey will be repeated to enable the calculation of incidence rates of SARS-CoV-2 infection in the study prisons as well as the proportion of positive tests of SARS-CoV-2 infection among residents and staff within the selected prisons.

Phase 2 comprises an enhanced outbreak investigation in 3 prisons in which a SARS-CoV-2 outbreak is declared. An outbreak is defined as two or more cases of SARS-CoV-2 that are epidemiologically linked [16].

### Setting and Site Selection

This study will be conducted in prisons across England. The sample of 28 prisons will be selected to be as representative of the closed prison estate as possible with regard to important features. These features are prison function and security category, geographical area, whether or not a COVID-19 outbreak was previously reported in that prison, staff numbers, resident population, and the proportion of the population classified as “older residents.” Operational factors, including capacity to undertake whole prison testing, will also be given consideration. In phase 1, all prisons will be selected. In phase 2, 3 prisons from the sample of 28 will be included when an outbreak is declared.

When rounds 1 and 2 of antigen testing have finished, if an outbreak of COVID-19 is declared by the local health protection team in any prison in England, further antigen testing of all prisoners and staff will take place as soon as is feasible after the outbreak has been declared. Testing will be repeated 7 days later and again at 28 days after the first test. This periodicity will give detailed information on outbreak dynamics. Staff will also be tested for antibodies to SARS-CoV-2 12 weeks after the start of the outbreak.

In both phases, the governor of each study prison will appoint a member of custodial staff to be the single point of contact (SPOC) to support the research project within the prison. The SPOC will be there to support staff and residents, and answer any questions or concerns they may have about the process.

### Sampling and Sample Size

**Figure 1:**
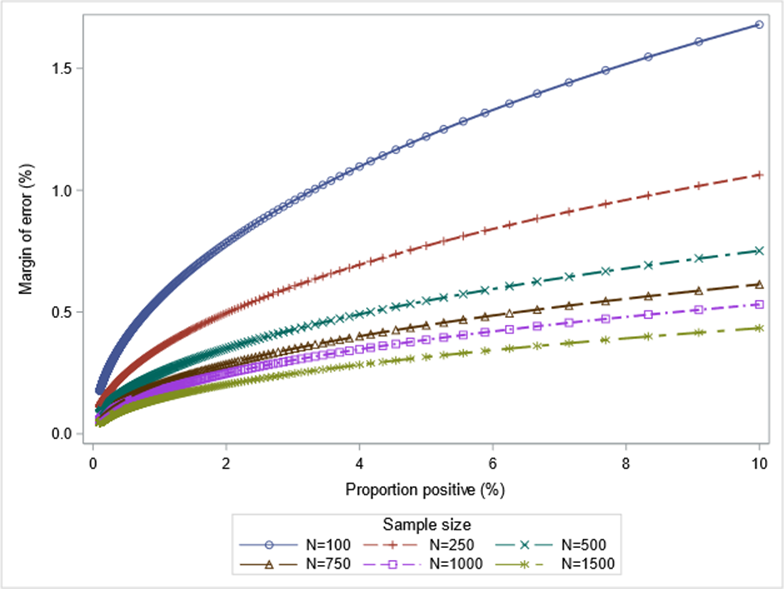
Impact of sample size on precision for various estimates of the proportion of positive tests.

For the primary objective of estimating the proportion of positive tests of SARS-CoV-2 infection within the selected prisons, the precision (margin of error) that various sample sizes (assuming 80% response) provide around various estimates of infection rates is illustrated in Figure 1.

The sample sizes (N) represent a range of individual prison population sizes from the smallest participating prison (approximately 100 prisoners) to the largest (approximately 1500 prisoners). For a given proportion of positive tests and sample size (N), the expected margin of error corresponds to the expected half-width of the 95% CI associated with the point estimate of the proportion of positive tests obtained using an exact binomial test. As illustrated, the precision of the SARS-CoV-2 positivity estimates in participating prisons is expected to be smaller for larger prisons. The 2 rounds allow for the secondary objective, the incidence of SARS-CoV-2, to be estimated; however, there will be limited power to detect small incidence rates.

### Study Team

The team comprises individuals with expertise in epidemiology and statistics, infectious diseases, public health, the prison system, and the conduct of research in prisons.

### Participant Eligibility and Recruitment

For all testing rounds in each participating prison (whether phase 1 or 2), all residents and staff present on the first day of data collection in that prison (the census date) are eligible to be included in the study (Textbox 1).

**Study inclusion and exclusion criteria for antigen testing.**

**Residents**

***Inclusion criteria***

- Age 16 years or older; male or female

- Currently a resident in the study prison

- Is willing and able to give informed voluntary consent for participation in the study

- Does not pose a security risk to the research team (risk assessed by custodial staff)

***Exclusion criteria***

- Is unwilling or unable to give informed voluntary consent for participation in the study

- Poses a security risk to the research team (risk assessed by custodial staff)

**Staff**

***Inclusion criteria***

- Age 18 years or older; male or female

- Currently employed to work in one of the study prisons

- Is willing and able to give informed voluntary consent for participation in the study

***Exclusion criteria***

- Is unwilling to give informed voluntary consent for participation in the study

- Shielding or long-term absence for the duration of the pandemic

### Study Variables

#### Data Collected From Imprisoned People

Prisoners will be asked to complete a paper questionnaire, and other data will be extracted by prison administrators for each individual from a secure prison information system (prison-National Offender Management Information System [p-NOMIS]). A summary of the data collected is provided in Table 1.

**Table 1:** Data collected from imprisoned people.

Data item	Data source	Time captured		
		Round 1 of antigen testing	Round 2 of antigen testing	Round 3 of antigen testing
Age	p-NOMIS^a^	Yes^b^	No^c^	Yes
Sex	p-NOMIS	Yes	No	Yes
Ethnicity	p-NOMIS	Yes	No	Yes
Time in the current prison	p-NOMIS	Yes	No	Yes
Place or residence prior to prison entry	p-NOMIS	Yes	No	Yes
Prison movements in the last 14 days	p-NOMIS	Yes	Yes	Yes
Current location	p-NOMIS	Yes	Yes	Yes
Shielding	Q^d^/p-NOMIS	Yes	Yes	Yes
Comorbidities	Q	Yes	No	No
Obesity	Q	Yes	No	No
Smoking status	Q	Yes	No	No
Symptoms for COVID (on testing day)	Q	Yes	Yes	No
Contact with COVID	Q	Yes	Yes	No
Past history of COVID	Q	Yes	Yes	No
Mental well-being	Q	No	Yes	No

^a^p-NOMIS: prison-National Offender Management Information System.

^b^Yes: data collected.

^c^No: data not collected.

^d^Q: paper questionnaire.

#### Data Collected From Staff

All data will be obtained from a self-complete questionnaire with the exception of data in an outbreak and second round of antibody testing if all staff are invited. These will be provided by the prison linked to the staff member’s number (not name). A summary of the data collected is provided in Table 2.

**Table 2:** Data collected from prison staff.

Data item	Time captured		
	Round 1 of antigen testing	Round 2 of antigen testing	Round 3 of antigen testing and antibody testing
Age	Yes^a^	No^b^	Yes
Sex	Yes	No	Yes
Ethnicity	Yes	No	Yes
Professional role	Yes	No	Yes
Area of residence	Yes	No	No
Occupations of household	Yes	No	No
Symptoms	Yes	Yes	No
Contact with COVID	Yes	Yes	No
Past history of COVID	Yes	Yes	No
Test history for COVID	Yes	Yes	No
Comorbidities	Yes	No	No
Smoking	Yes	No	No
Mental well-being	No	Yes	No
Vaccination status (against SARS-CoV-2)	No	No	Yes

^a^Yes: data collected.

^b^No: data not collected.

#### Prison-Level Data

The operational SPOC will be asked to obtain data outlined in Table 3 and complete an electronic questionnaire provided by the research team. The research team will obtain further information from Her Majesty’s Inspectorate for Prisons reports on information, such as concerns about the fabric of the building or sanitation issues. A summary of the data collected is provided in Table 3.

**Table 3:** Data collected about each participating prison.

Data item	Data source	Time captured		
		R1	R2	R3
Name of prison	Q^a^	Yes^b^	No^c^	No
Security category (if appropriate)	Q	Yes	No	No
Functional designation	Q	Yes	No	No
HMPPS^d^ region	Q	Yes	No	No
In-use certified normal accommodation	Q	Yes	No	No
Current population	Q	Yes	Yes	Yes
Percentage aged 50 years or more	Q	Yes	No	No
Percentage from Black, Asian, and minority ethnic groups (with breakdown)	Q	Yes	No	No
Number of staff directly employed	Q	Yes	No	No
Number of staff indirectly employed and by which contractor	Q	Yes	No	No
Year the prison was built (and additions)	Q	Yes	No	No
Geographical layout	Map/photo	Yes	No	No
Number of wings	Q	Yes	No	No
Number of landings on each wing	Q	Yes	No	No
Number of cells on each landing	Q	Yes	No	No
Number of single cells	Q	Yes	No	No
Number of multiple occupancy cells	Q	Yes	No	No
Number of cells currently occupied by two or more people	Q	Yes	Yes	Yes
Number of cells with the following types of in-cell sanitation: in-cell washbasin, in-cell toilet plus washbasin, in-cell toilet plus washbasin plus shower	Q	Yes	No	No
Sanitation problems reported in HMIPRs^e^	HMIPRs	Yes	No	No
Problems reported by HMIP^f^ regarding the fabric of the building	HMIPRs	Yes	No	No
Ventilation system (air conditioning/natural air flow from windows/ceiling fans)	Q	Yes	No	No
Date created, capacity, and current occupancy of the following 3 units: protective isolation unit, reverse cohorting unit, and shielding unit	Q	Yes	Yes	Yes
Regime changes (eg, reinstatement of visits)	Q	No	Yes	Yes

^a^Q: electronic questionnaire.

^b^Yes: data collected.

^c^No: data not collected.

^d^HMPPS: Her Majesty’s Prison and Probation Service.

^e^HMIPRs: Her Majesty’s Inspectorate for Prisons reports.

^f^HMIP: Her Majesty’s Inspectorate for Prisons.

### Study Processes: Testing

All data collection will be done within the prison. Private contractors will undertake the testing. In each prison, there will be a team of 6 to 10 testing staff fully trained in testing procedures by their employer and trained in the study procedures and ethical practice by the University of Southampton. Each tester will be escorted around the prison by a member of the custodial staff, who will open the cell door to each participant. At the first contact, the tester will take written consent for the whole study from the participant and collect the completed paper questionnaire. At subsequent testing rounds, verbal consent will be obtained. In phase 1, participants will complete a self-swab of their nose and throat as instructed and observed at 2 m by the tester. In phase 2, participants will self-swab twice. They will provide a nasal swab for testing using a lateral flow device and a nasopharyngeal swab for polymerase chain reaction testing. A member of the health care team will swab any residents with a physical disability that makes it difficult for them to self-swab. For later rounds of testing in an outbreak situation, the testing process will be supervised as previously, by externally contracted staff, or by the prison health care team, dependent on local circumstances.

A private provider, commissioned by HMPPS to provide occupational health services to prison staff, will take a swab of their nose and throat or, alternatively, staff will swab their own nose and throat if the occupational health provider is unable to do this. For later rounds of testing in an outbreak situation, staff will self-administer the test as part of HMPPS’s ongoing weekly testing program. They will also self-administer a second nasal swab that will be used by the testing team to test using a lateral flow device. They will receive an information sheet and a consent form to enable their data to be shared with the study team.

The nose and throat swabs will be couriered to an accredited laboratory where swabs will be tested for the presence of SARS-CoV-2 using reverse transcription polymerase chain reaction (RT-PCR). Before residual material is discarded, if the virus is detected, the material will be couriered securely to a COVID-19 Genomics UK Consortium study laboratory for genomic sequencing of the virus in accordance with the COVID-19 Genomics UK Consortium study protocol [16]. The nasal swabs will be tested on site using Innova lateral flow devices; these provide a result within 30 minutes.

Staff who participate in round 1 or 2 of antigen testing will be invited to participate in antibody testing. Following the second round of testing, the SPOC will issue participating staff with a home testing kit for antibody testing.

### Data Collection: Instruments and Sources

Participating residents will complete a brief paper questionnaire that asks about medical risk factors for severe COVID-19, current COVID-19 symptoms, and symptoms in the past 14 days in line with other COVID studies in England. In the second round, the short version of the Warwick Edinburgh Mental Well-Being Scale (WEMWBS), a well-validated measure of mental well-being [17], will be included in the questionnaire. Further information about residents’ age, ethnicity, location, and movements within the prison will be obtained by the SPOC (or appointed administrator) from the secure p-NOMIS. In an outbreak situation, questionnaires will not be completed, but data will be obtained on residents’ ethnicity, age, and sex.

Each time they are tested, each participating staff member will complete a short online questionnaire providing details of medical risk factors for severe COVID-19, current COVID-19 symptoms, and symptoms in the past 14 days. In the second round, the WEMWBS will be included in the staff questionnaire too. In an outbreak situation, questionnaires will not be completed, but data will be obtained on the staff members’ ethnicity, age, and sex.

The SPOC will gather information about the prison for the study team, including geographical layout, age, ventilation systems, sanitation, numbers of residents and staff, and what infection control measures have been implemented. The study team will obtain data on the prevalence of SARS-CoV-2 in the community from the ongoing Office of National Statistics Community Survey [18].

### Data Management and Analysis

Data will be stored and handled at the University of Southampton and PHE. A pseudonymized database will be created using unique participant IDs.

Summary statistics (N, proportion, mean, SD, and IQR) will be obtained to summarize the prison and participant characteristics. Tables will display the number of participants in each of the 2 surveys, the number of new participants (eg, new residents), and the number of individuals withdrawn/lost to follow-up from the previous survey (eg, withdrawn consent or left the prison).

For the primary objective, simple proportions of individuals testing positive for SARS-CoV-2 following RT-PCR of samples (based on nose and throat swabs) will be calculated for each prison population of staff and prisoners separately. The proportion of positive individuals with symptomatic infection, defined as “yes” to any of the listed symptoms in the questionnaire, will also be calculated for each prison.

Stratified proportions will be estimated to understand the variation in the prevalence of infection among different prison characteristics and different populations (eg, the strata of staff/resident population; prison type; participant age, gender, and ethnicity; current location in prison [prison population only]; and staff role [staff population only]).

Incidence rates within each prison will be calculated. Variation in incidence rates among different prison characteristics and different populations will be explored by estimating stratified incidence rates within the strata of staff/prisoner population; prison type; participant age, gender, and ethnicity; current location in prison (prisoner population only); and staff role (staff population only).

To examine the individual, institutional, system, and community factors associated with SARS-CoV-2 infection within prisons, generalized linear regression models will be fitted to adjust prevalence and incidence rates for relevant hypothesized risk factors. The list of individual-level risk factors includes participant age, gender, ethnicity, and existing comorbidities; and staff role and responsibilities (for staff analyses only). Institutional factors include in-cell sanitation (for resident analyses only), establishment age, ventilation type, use of compartmentalization measures, and regional location. All point estimates will be provided with 95% CIs.

The proportion of staff who test antibody positive at week 6 (overall and according to whether they are prisoner-facing or not) will be calculated for each prison.

The WEMWBS will be scored according to the user guide [19]. The mean scores from the short WEMWBS will be presented for residents and staff, and their associated demographic factors.

Imputation methods may be employed depending on the level of completeness for any specific analysis.

### Ethical Considerations

This is a time-pressured study that has high-level UK Government support. All research within prisons has currently been stopped because of the COVID-19 pandemic, and researchers are not allowed into prisons. This study has been allowed to go ahead using professional testing staff who will be wearing appropriate personal protective equipment (PPE). The University of Southampton team will be training (remotely) these staff members to inform them about the study, with a particular emphasis on understanding the principles of ethical research conduct and the specificities of this in a prison setting. The Southampton research team will act as an ongoing resource for advice about the conduct of the study for all testing staff.

The main study-specific ethical consideration is the inclusion of prisoners, a group regarded as vulnerable in international guidance on the ethical conduct of health research. In addition, some of these prisoners are 16 and 17 years old. There are a number of key considerations as presented below.

#### Informed Voluntary Consent

The participant information sheet (PIS) for residents gives a detailed account of the aims of the research and what it will involve, highlighting that participation is entirely voluntary and that nonparticipation will not have any adverse effects for participants (specifically, it will not affect care or parole). Participants will have the opportunity to discuss the research and ask questions with a number of people. There will be 2 SPOCs in each prison who will be available to discuss any issues with individuals. Each prison has also been asked to identify “peer mentors,” prisoners who will familiarize themselves with the study and receive written materials prepared by the University of Southampton. They will be able to discuss issues with their fellow prisoners. Finally, the tester is an independent person who is not allied to the prison. It is important that consent to participate in the research is not undertaken by the prison officers or other members of the prison staff to ensure that the decision is not influenced by someone in a position of authority. This is important as prisons are widely perceived to be a coercive environment, and all prisoners must feel able to decline participation should they wish. For those who do want to participate, the consent form will be used to ensure that participants have understood the information provided and are aware what participation involves.

The level of literacy in the prison population is lower than that in the general population, and so, the consent form and PIS are suitable for people with limited literacy skills.

The prison health care team will be asked to draw up a list of prisoners who should be excluded as they are unable to give informed consent because of mental illness. These particularly vulnerable individuals will be excluded from the study.

Staff might also feel pressurized to participate because of the hierarchical nature of the prison service. The PIS will emphasize that participation is voluntary, and an occupational health professional, independent of the prison service, will discuss the study with them and ensure consent is provided voluntarily. Nonparticipation will not affect their work in any way.

#### Right to Withdraw

The PIS for each participant group provides details of how to withdraw from the study. Withdrawal from the study does not affect care or treatment in the prison for the prisoner or staff member.

#### Risk of Harm

The risk of harm has been assessed by the study team as low. The questionnaire does not require sensitive information, and the throat and nose swabs are unpleasant but not invasive. The blood test is a simple standardized procedure conducted by trained staff who will adopt the necessary precautions.

#### Confidentiality

Only the research team (named on the information sheet) and authorized personnel from the study team will have access to participant data. All information gathered will be stored in accordance with General Data Protection Regulation (GDPR) guidelines.

In this study, complete confidentiality of the antigen testing results is not possible. Prisoners who test positive will be isolated in accordance with government guidelines, and this will be apparent to staff and prisoners in their wing. Similarly, staff who test positive will have to isolate at home, and because they will be unable to come in to work, this will be apparent to colleagues. This will be made clear in the PIS.

#### Involvement of Young People Aged Under 18 Years

In the absence of law relating specifically to research, it is commonly assumed that the principle of “Gillick competence” can be applied to consent for research. This means that if a young person has sufficient understanding and intelligence to understand fully what is proposed and can use and weigh this information in reaching a decision, they can give consent. As their competence to understand is influenced by how the information is presented, we have ensured that the young person’s chances of understanding what is involved in the study are maximized by developing an information sheet that uses straightforward language and has been formally assessed for ease of reading. In addition, the young people will have the opportunity to discuss the study with fully briefed staff members and SPOCs.

#### Infection Risk to Professionals Involved in Conducting the Study

There is a risk that if those participating in the study are infected with SARS-CoV-2 (either staff or prisoners), they might infect those testing them. Equally, participating prisoners and staff might be infected by the testing staff. This risk will be minimized by the use of PPE and observing social distancing rules.

All residents and staff who are willing to participate will provide informed consent. Approval for this study has been granted by the University of Southampton Research Integrity Group (number 57844) and by the Health Research Authority and Health and Care Research Wales (IRAS project ID: 285534; REC reference: 20/NW/0320). These are the bodies that provide ethical approval for this sort of study in England.

### Study Timeline

The study timeline is as follows: antigen testing in round 1 from July 20 to August 22, 2020; antigen testing in round 2 from September 1 to October 2, 2020; antigen testing in round 3 from February 11 to March 31, 2021; antibody testing from October 5, 2020, to March 31, 2021; analysis of data from August 1, 2020, to December 31, 2021; and dissemination of findings from April 1, 2021, to April 1, 2022.

## Results

We secured all relevant ethical approvals on July 7, 2020, and data collection was started on July 20, 2020. Data collection will end on May 31, 2021. As of May 2021, we have enrolled 4192 staff members and 6496 imprisoned people in the study. Data analysis has started, and we expect to publish the initial findings in autumn/winter 2021/2022.

## Discussion

### Study Strengths

There are approximately 12 million people imprisoned worldwide [20], and to the best of our knowledge, this is the first national study to examine the incidence and prevalence of SARS-CoV-2 in prisons in the world. COVID-19 in prisons is an important public health issue that has implications for not only those who live and work in prisons, but also the wider society. Prisons are high-risk settings for the transmission of infectious diseases, and this study will provide data important for practice, public health, and policy making across prisons in an entire country during the COVID-19 pandemic. There needs to be an in-depth understanding of the transmission of SARS-CoV-2 within prisons, from the community to prisons and from prisons to the community, in order to inform effective measures to tackle the virus within this unique setting.

With an increasing focus on the mental health implications of the pandemic, the findings from this study will quantify mental well-being in a sample of participants (both residents and staff) using a tool that has been widely validated in the community and in imprisoned people. This will provide important information to enable infection control measures to be appropriately targeted, balancing interventions to prevent infection with the needs of residents and staff to maintain physical and mental health. This unique combination of complimentary data on the epidemiology of SARS-CoV-2 within prisons and on mental well-being will be invaluable in helping adopt a more holistic approach to the implementation of infection control measures in prisons, ensuring that any measures adopted can, as far as is practicable, take into account the impact on the mental well-being of imprisoned people. This is particularly important because of the long-standing nature of this pandemic and can inform health security challenges in the future.

The findings from the study will be made widely available through open-access publications to enable the benefits of the knowledge gained to extend beyond national boundaries. Furthermore, the expertise gained by the research team from the conduct of this unique research in prisons will be shared with other countries using platforms such as the Worldwide Prison Health Research and Engagement Network.

### Study Limitations

The study does however present huge logistical challenges. Testing up to 10,000 prison staff and 20,000 prison residents at 2 different time points is a considerable undertaking. Researchers are currently not allowed into prisons, and therefore, testing teams, trained in antigen testing for SARS-CoV-2 and the use of PPE, must be deployed. They will need security clearance to enable them to go into prisons. They must be trained remotely in ethical research conduct to enable them to receive informed voluntary consent from participants. This is particularly important as prison residents are classified as “vulnerable” participants according to international ethical guidelines [21] and many challenges in the ethical conduct of prison research have been identified prior to the pandemic, which adds complexity to the processes [22].

The use of information technology in the study is limited because of the prison environment. Questionnaires for residents will be on paper; the use of electronic devices by resident participants is not permitted. This creates delays in receiving data in a form that can be readily analyzed and the potential for data losses through lost questionnaires, transcription errors, etc. Staff will complete an online questionnaire in the prison at the time of testing, but ensuring all staff have access to a computer presents logistical challenges in an environment where the use of information technology, even by staff, is greatly restricted.

The study was developed at great speed and in a fraction of the time that a study of this nature would ordinarily require. This resulted in a level of patient and public involvement (PPI) that was significantly lower than usual for the research team. Furthermore, during the protocol development phase, the research team was not directly working with prison staff or residents. HMPPS liaised directly with the key staff union, the Prison Officers Association, and the governor of each prison was delegated the task of working with resident groups. PPI representation on the independent study steering group was, however, highly engaged and a source of valuable insights.

### Potential Study Impact

The COVID-19 in Prison Study presents an important opportunity to gather data in a unique high-risk setting from the whole population (residents and staff alike). It will provide important epidemiological data to develop our understanding of the transmission dynamics of SARS-CoV-2 in prisons and provide preliminary data on the mental well-being of staff and residents. The study will thus provide broad and more holistic data to inform public health policy and practice in prisons during the COVID-19 pandemic.

## Abbreviations

HMPPS: Her Majesty’s Prison and Probation Service
NHS: National Health Service
PHE: Public Health England
PIS: participant information sheet
p-NOMIS: prison-National Offender Management Information System
PPE: personal protective equipment
PPI: patient and public involvement
RT-PCR: reverse transcription polymerase chain reaction
SPOC: single point of contact
WEMWEBS: Warwick Edinburgh Mental Well-Being Scale
WHO: World Health Organization
